# Inhibitory interneurons enable sparse code formation in a spiking circuit model of V1

**DOI:** 10.1186/1471-2202-13-S1-P148

**Published:** 2012-07-16

**Authors:** Paul D King, Joel Zylberberg, Michael R DeWeese

**Affiliations:** 1 Redwood Center for Theoretical Neuroscience, University of California, Berkeley, California, 94720, USA; 2 Helen Wills Neuroscience Institute, University of California, Berkeley, California, 94720, USA; 3 Department of Physics, University of California, Berkeley, California, 94720, USA

## 

Sparse coding accounts for several physiological properties of primary visual cortex (V1), including the shapes of simple cell receptive fields and the highly kurtotic firing rates of V1 neurons [1]. Current spiking network models of pattern learning [2] and sparse coding [3] require direct inhibitory connections between the excitatory simple cells, in violation of Dale's Law which states that neurons can either excite or inhibit but not both. At the same time, the computational role of inhibitory neurons in cortical microcircuit function has yet to be fully explained.

**Figure 1:**
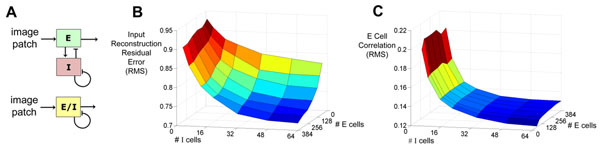
**A**. Circuit diagram of our spiking network with separate excitatory (E) and inhibitory (I) neural populations (top) compared to current single population models (bottom). This network was simulated with different numbers of excitatory and inhibitory cells. **B**. Adding inhibitory cells to the network differentiates the receptive fields and decreases image reconstruction error during learning. **C**. This error reduction is caused by decreased correlations among the excitatory neurons that are collaborating to form a sparse representation of the visual input. The network was trained on 8x8 image patches (64 pixels) drawn from whitened natural images. Excitatory neuron counts (# E cells) ranged from 64 to 384 (1x to 6x overcomplete). Inhibitory neuron counts (# I cells) ranged from 3 to 64 (.05x to 1x overcomplete). We find that reconstruction errors are roughly constant for populations of interneurons that are at least ~20% of the size of the total population, assuming the total neural population is at least 4x overcomplete relative to the input. This is consistent with the 80/20 ratio of excitatory-to-inhibitory neurons observed in visual cortex.

Here we show that adding a separate population of inhibitory neurons to a recently proposed model of V1 [3] not only brings spiking network models of sparse coding in line with Dale’s Law, but it also predicts excitatory-to-inhibitory neuron ratios and explains how inhibitory neurons may function computationally. When trained on natural images, this excitatory-inhibitory spiking circuit learns Gabor-like receptive fields as found in V1 using spiking neurons and synaptically local plasticity rules. The inhibitory cells enable sparse code formation using a novel learning rule by collaboratively discovering and suppressing correlations within the excitatory population (Figure 1). The model predicts that only a small number of inhibitory cells is required relative to excitatory cells, matching physiological ratios observed in primary visual cortex.
